# Real-time and on-line monitoring of ethanol fermentation process by viable cell sensor and electronic nose

**DOI:** 10.1186/s40643-021-00391-5

**Published:** 2021-05-11

**Authors:** Yao Feng, Xiwei Tian, Yang Chen, Zeyu Wang, Jianye Xia, Jiangchao Qian, Yingping Zhuang, Ju Chu

**Affiliations:** State Key Laboratory of Bioreactor Engineering, East China University of Science and Technology, 130 Meilong Road, P. O. Box 329#, Shanghai, 200237 China

**Keywords:** Ethanol, On-line, Viable cell sensor, Electronic nose

## Abstract

In this study, introduction of a viable cell sensor and electronic nose into ethanol fermentation was investigated, which could be used in real-time and on-line monitoring of the amount of living cells and product content, respectively. Compared to the conventional off-line biomass determination, the capacitance value exhibited a completely consistent trend with colony forming units, indicating that the capacitance value could reflect the living cells in the fermentation broth. On the other hand, in comparison to the results of off-line determination by high-performance liquid chromatography, the ethanol concentration measured by electronic nose presented an excellent consistency, so as to realize the on-line monitoring during the whole process. On this basis, a dynamic feeding strategy of glucose guided by the changes of living cells and ethanol content was developed. And consequently, the ethanol concentration, productivity and yield were enhanced by 15.4%, 15.9% and 9.0%, respectively. The advanced sensors adopted herein to monitor the key parameters of ethanol fermentation process could be readily extended to an industrial scale and other similar fermentation processes.
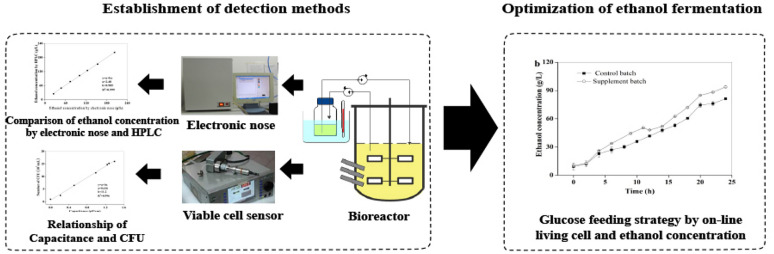

## Highlights

- Living cells were on-line monitored by viable cell sensor.
- Ethanol concentration was on-line monitored by electronic nose.
- A glucose feeding strategy was developed to improve ethanol production.

## Introduction

With the increasing crises of environmental pollution and global warming caused by the excess use of fossil fuels, biofuels are regarded as the most potential renewable bioenergy to deal with global climate change and energy security (Burphan et al. 2018). Mixing a certain proportion of ethanol into gasoline can alleviate the energy problem and reduce the emission of pollutants to some extent (Jagtap et al. 2019). Replacing fossil fuels with bioethanol can cut carbon dioxide emissions from cars by 90% (Khongsay et al. 2012). Therefore, it is very necessary to increase the yield of fuel ethanol. However, it still has some disadvantages in terms of economy (Taiwo et al. 2018). There are many factors that affect the economy of bioethanol, such as the selection of raw materials, the control of bioprocess, the treatment of by-products and so on. Increasing the conversion rate of substrates can significantly improve the economy of ethanol fermentation, so that the development of efficient ethanol fermentation technology is the key to achieve cost-effective production.

On-line detection of process parameters plays an important role in the recognition of fermentation process characteristics. Especially, the real-time acquisition of key parameters can provide a basis for on-line dynamic regulation. A variety of process regulation strategies have been developed to achieve efficient fermentation of ethanol by *Saccharomyces cerevisiae*, including alleviation of high concentration of substrates and product inhibition (Zhang et al. 2015; Ji et al. 2012), engineering for high-performance strains (Wu et al. 2020; Naghshbandi et al. 2019; Liu et al. 2019) and optimization of environmental conditions (Zhang et al. 2020; Liu et al. 2013). However, at present, the common control of industrial ethanol fermentation process is based on artificial experience and on-line detection parameters mainly involved in operating parameters, while key indexes including biomass, substrates, product and by-products, which are of great importance, were basically measured by off-line methods (Dekker et al. 2017). Although some advanced sensors have been introduced into the fermentation process in recent years, such as near-infrared spectroscopy (Grassi et al. 2013; Muncan et al. 2021; Vann et al. 2017), Raman spectroscopy (Hirsch et al. 2019; Jiang et al. 2020; Metcalfe et al. 2020), they were mainly adopted in laboratory scale. These sensors are expensive and generally beyond the tolerance of the manufacturer. They also have high requirements for the environment in which they are used, which the industrial environment cannot meet. The viable cell sensor can specifically characterize the number of living cells in the fermentation broth by measuring the capacitance value. It has been successfully applied to monitor the concentration of living cells in various fermentation processes (Kedia et al. 2013; Xiong et al. 2008; Wang et al. 2016; Zeiser et al. 1999; Kroll et al. 2017). The viable cell sensor was used to guide the supplementation of nutrients during the polyhydroxyalkanoates (PHAs) fermentation, which increased the yield of PHAs by 22.0% (Li et al. 2014). On the other hand, the on-line detection of product concentration is also crucial for the control of fermentation process, which can be used to determine the fermentation state, feeding time, fermentation end point, etc. Electronic nose can quantitatively analyze the content of specific components in the off-gas through the sensitive films, so as to realize the on-line detection of volatile substances in the fermentation broth (Wisniewska et al. 2015; Buratti et al. 2011; Mendez-Rodriguez et al. 2020). Real-time monitoring of n-propanol concentration was realized during industrial erythromycin fermentation by electronic nose, and then the feedback control strategy of n-propanol concentration was developed to achieve a maximum erythromycin yield of 8500 U/mL (Zhao et al. 2016). Similar with the n-propanol, ethanol is also a volatile substance, so it is theoretically feasible to be on-line monitored by electronic nose. Buratti et al. (2011) used electronic nose to detect volatile components in wine fermentation, and Park et al. (2017) used mass spectrometry (MS) with electronic nose instead of gas chromatography to analyze the ethanol content in soy sauce. Notably, these applications of the electronic nose were conducted by off-line method, which are time-consuming and labor-intensive.

In this study, living cell sensor and electronic nose were introduced into the ethanol fermentation process, which could on-line detect living cells and ethanol concentrations, so as to determine the metabolic state of the cells and develop effective process regulation strategy to achieve cost-effective production. By establishing the mathematical models between the capacitance value and the number of living cells as well as between the signal of sensitive responded channel in the electronic nose and the ethanol concentrations, the on-line monitoring of key index parameters was realized. On this basis, the on-line feeding strategy of glucose was developed under the guidance of the quantitative changes of living cells and ethanol concentration in the process, so as to effectively improve the ethanol fermentation performance.

## Materials and methods

### Strain, media and culture conditions

The strain used in this study was *S. cerevisiae* B1, which was preserved by the National Center of Bio-Engineering and Technology (Shanghai). The basal seed and fermentation media contained (g/L): KH_2_PO_4_ 10, MgSO_4_ 0.5, Yeast Extract 5, CaCl_2_ 0.1, (NH_4_)_2_SO_4_ 5. The initial glucose concentrations in seed, control batch and supplement batch were 40 g/L, 200 g/L and 100 g/L, respectively. In terms of supplement batch, another 100 g/L glucose would be added when the capacitance value of fermentation broth showed a downward trend and the corresponding ethanol concentration also showed a slight decrease. The seed culture was carried out in a 250-mL shake-flask containing 100 mL working volume at 220 rpm and 30 °C for 14 h (OD_600_≈8) and then inoculated into the fermentation medium at 20% inoculum volume. For 5-L bioreactor (Shanghai Guoqiang Bioengineering Equipment Co., Ltd., China) culture, the initial volume of 2.4 L with an inoculum of 0.6 L was cultured at 30 °C and 150 rpm for 24 h with no aeration.

### Factors influencing the response of electronic nose

**Fig. 1:**
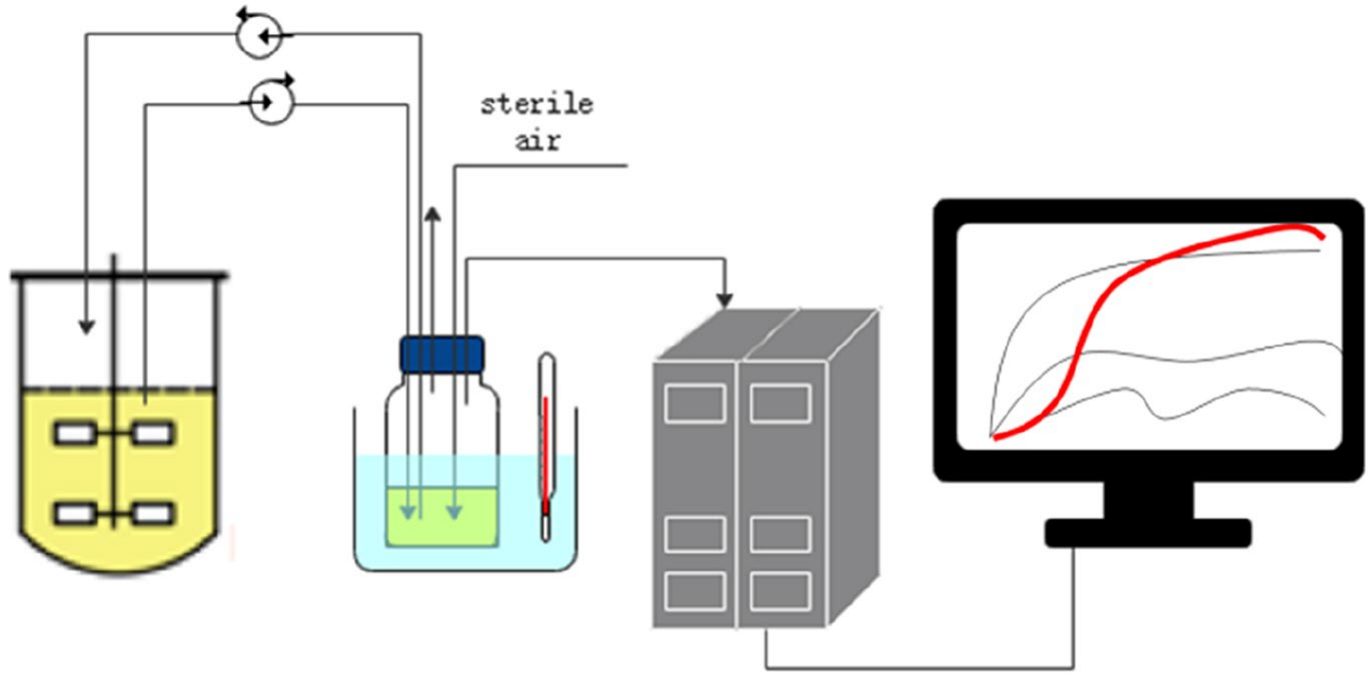
Schematic diagram of electronic nose detecting system for real-time and on-line monitoring ethanol concentration in fermentation process

A corresponding experimental device was developed for the non-aerated fermentation process (Fig. 1). The effects of deionized water, culture medium and fermentation broth on the detection of ethanol by electronic nose were studied. Moreover, the effects of different liquid loading volumes in the glass bottle (50, 100, 150, 200 mL) and different aeration (1, 2, 3, 4 L/min) were explored as well.

### Glucose feeding strategy

In a 5-L bioreactor, the initial glucose concentration was 100 g/L, the inoculating amount was 20%, the culture temperature was 30 °C, and the stirring speed was 150 rpm. The changes of capacitance value and ethanol content in the fermentation broth were detected on-line in real-time by a viable cell sensor and electronic nose, respectively. When the capacitance value and the electronic nose signal decreased continuously within 60 min, high concentration glucose solution (800 g/L) was added to make the glucose concentration in the fermentation broth at about 100 g/L after supplementation.

### Analytical methods

Optical density (OD) is obtained by collecting 1 mL sample from the fermentation broth every 2 h, which is diluted for a certain multiple and tested with a spectrophotometer at a wavelength of 600 nm.

Dry cell weight (DCW) was harvested by collecting 8 mL samples from the fermentation broth every 2 h and added to an empty tube. The sample was centrifuged at 4 °C and 4000 rpm for 5 min. The supernatant was discarded, and then the cells were washed for one time with 8 mL deionized water. After centrifugation, the cells were dried to constant weight at 70 °C for 24 h.

Colony forming units (CFU) was counted through 1 mL sample collected from the fermentation broth every 2 h. The broth was diluted to an appropriate multiple with sterile water, and then 40 μL sample was spread on a plate and cultured in a 30 °C incubator for 48 h.

The concentration of residual glucose was measured using an enzymatic bio-analyzer (SBA-40C, Shandong Academy of Sciences, China).

A 20 mL sample was collected from the fermentation broth every 2 h and centrifuged at 4000 rpm for 5 min. After dilution of the supernatant by a certain multiple, ethanol was determined by high-performance liquid chromatography (HPLC, Agilent 1100, America) and refractive index detectors at a column temperature of 50℃. The mobile phase was 10 mmol/L H_2_SO_4_ and the flow rate was 0.4 mL/min.

The ethanol productivity and yield were calculated based on Eqs. (1) and (2):

1 $$\mathrm{Productivity}\left(\mathrm{g}\cdot {L}^{-1}\cdot {h}^{-1}\right)=\frac{Final\, ethanol\, content-initial\, ethanol\, content+sample \,loss\, ethanol}{3\times 24},$$

2 $$\mathrm{Yield}\left(g/g\right)=\frac{Ethanol \,production(g)}{Glucose\, consumption(g)}$$

### Capacitance measurement

Viable cell sensor 220 (METTLER TOLEDO) was directly connected to a 5-L bioreactor, and the channel of yeasts/fungi fermentation was selected to on-line detect the capacitance value of the fermentation broth. The sampling interval is 30 min.

### Application of electronic nose to the real-time and on-line detection of ethanol concentration

The electronic nose was independently developed by East China University of Science and Technology (Zhao et al. 2016). The quantitative detection of different volatile components was realized through the changes of resistances by 16 sensitive channels. As shown in Fig. 1, the fermentation broth was pumped from the 5-L bioreactor to a 250-mL glass bottle through the peristaltic pump at the flow rate of 45 mL/min. Then the broth in the bottle was returned to the fermentation tank through another peristaltic pump, so that the loading volume in the bottle could be controlled at 100 mL constantly. 1 L/min of sterile air was injected into the bottle, and a stream of gas was extracted from the bottle and injected into the electronic nose automatically for real-time and on-line detection of ethanol concentration. According to Henry's Law, when the pressure of gas phase is relatively small, the vapor pressure of solute is proportional to the concentration of solute. The electronic nose sucked air above the 250-mL glass bottle into the test box with a miniature diaphragm pump at 25 mL/s and skimmed it over the surface of the sensitive membrane. Then the film sensors in each channel converted the concentration of the volatiles into certain electrical signal (Zhao et al. 2016). The time interval of measurement by electronic nose was set as 10 min.

In terms of the determination of detecting limit, ethanol-free deionized water was used to determine the fluctuation range of the electronic nose baseline. The measured standard error was adopted as noise and the detecting limit was set as the sample concentration corresponding to the average baseline plus 3 times of the noise. For the response range of ethanol concentration by electronic nose, the lower limit of the response value was the detecting limit, and the upper limit was the highest response value that the electronic nose channel could reach within the pre-setting range of ethanol concentration.

### Statistical analysis

All experiments were performed in triplicate and all data were presented as the mean with standard deviation. Statistical analysis was performed using one-way analysis of variance and Duncan's new multiple range test (*P* < 0.05) was used to test whether there was any significant difference among treatments.

## Results and discussion

### Application of the viable cell sensor in ethanol fermentation process

**Fig. 2:**
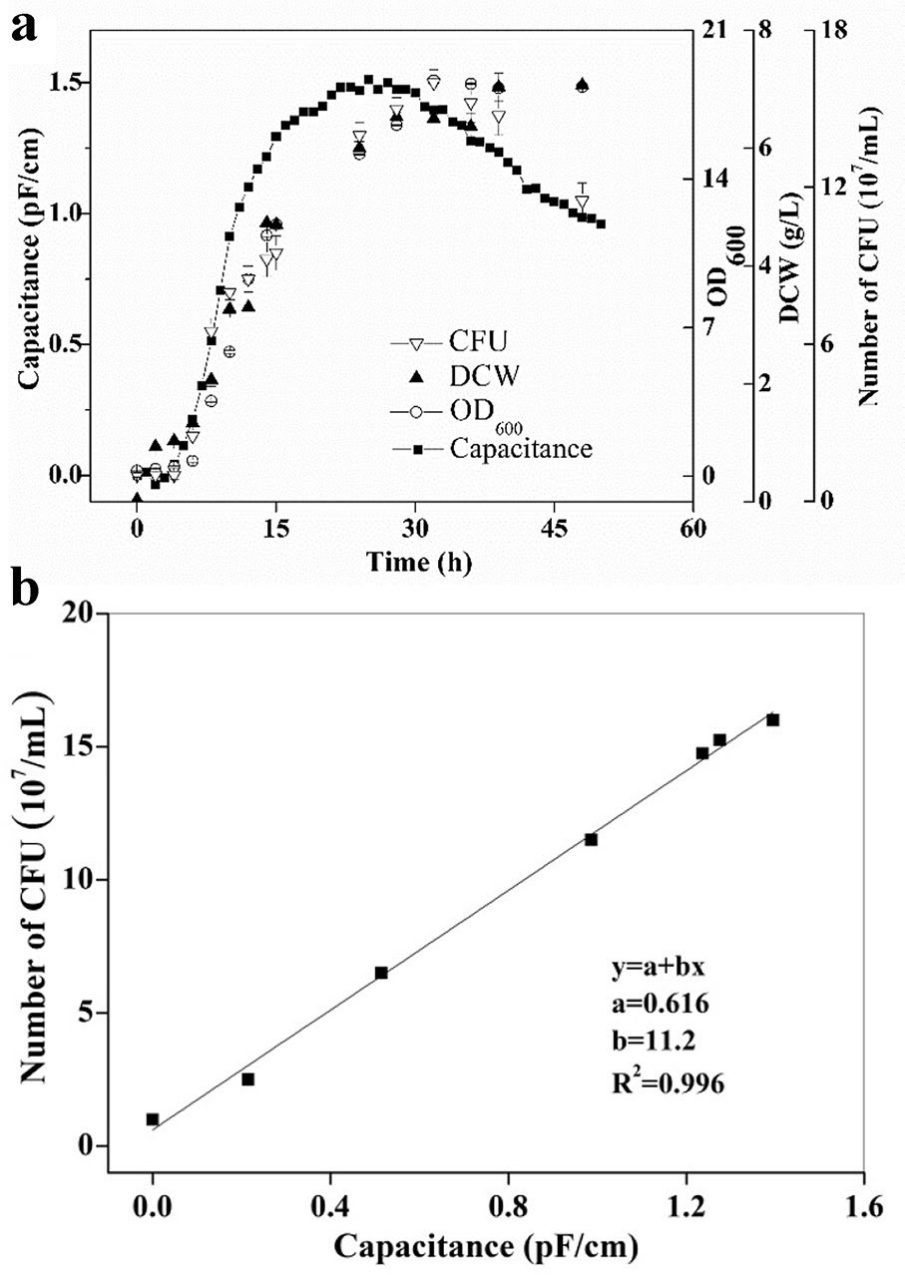
Time curve of capacitance value and OD_600_, DCW, CFU in a 5-L bioreactor (**a**), capacitance value and CFU (**b**)

Figure 2 illustrates the change curves of three regular off-line biomass determining values (OD_600_, DCW and CFU) and the capacitance values in fermentation broth during the ethanol fermentation process. It could be observed that within 8–32 h after inoculation, all the four parameters showed a significant increase with the similar trend. However, after 32 h, the variation trend showed great differences. The capacitance value and CFU value began to decline remarkably, while OD_600_ and DCW continued to maintain at high levels (Fig. 2a). By off-line measuring the glucose concentration, it indicated that the glucose has been as low as 5 g/L at 30 h, and exhausted at 32 h. At this point, the cell metabolism would be severely limited, and thus entered into the decline period. OD_600_ and DCW could not distinguish the living and dead cells during the determination, while the CFU value only represented the amount of living cells in the fermentation broth. Therefore, the trend of capacitance value could effectively and truly reflect the number of living cells. By fitting the values of capacitance, OD_600_, DCW and CFU, the results showed that the linear relationship between capacitance and CFU was the best, where *R*^2^ reached 0.996 (Fig. 2b). The Pearson correlation coefficients between capacitance value and OD, DCW, CFU are, respectively, 0.90, 0.90 and 0.94. Therefore, in the ethanol fermentation process, through on-line measuring the capacitance value to reflect the number of living cells, the physiological and metabolic characteristics of the cells could be further understood.

### Application of electronic nose in ethanol fermentation process

**Fig. 3:**
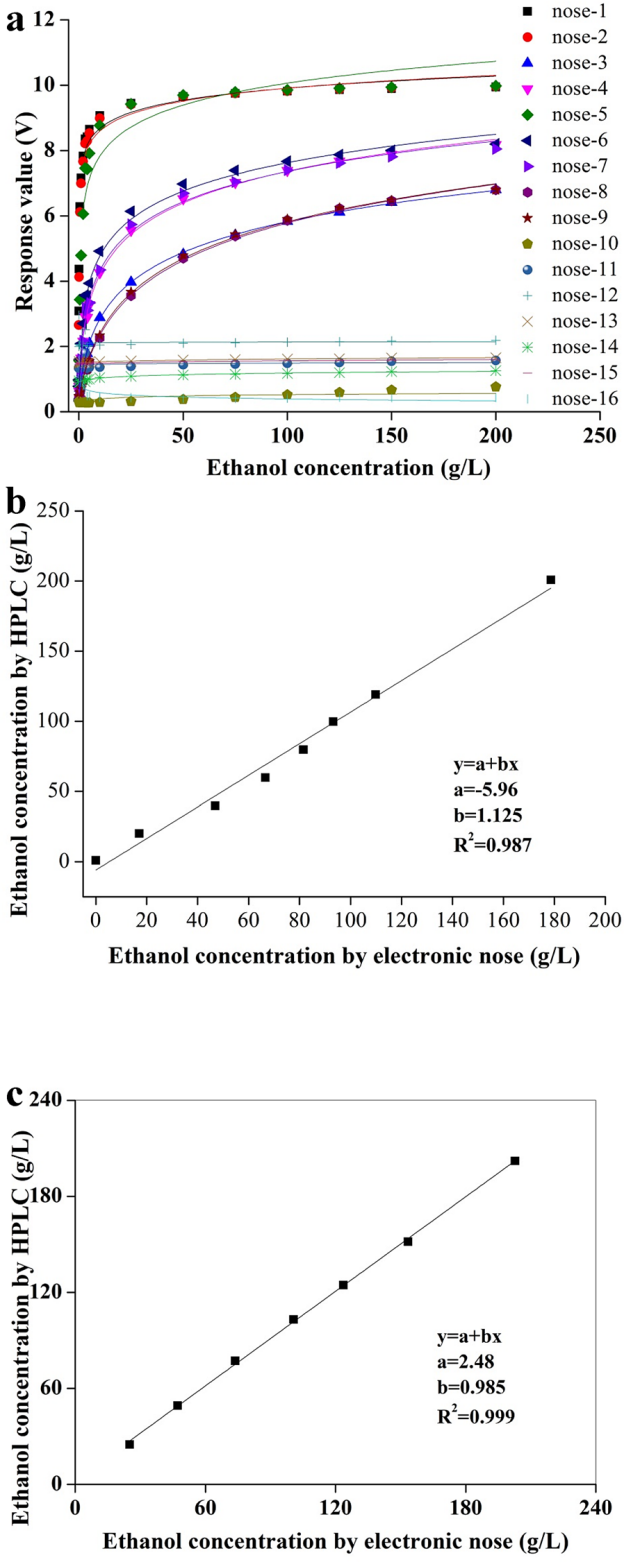
Response curves of each channel in electronic nose to different ethanol concentrations (**a**), relationship between the ethanol concentrations determined by electronic nose and by HPLC. Channel 4 (**b**), Channel 3 (**c**)

Ethanol solutions of different concentrations (0–200 g/L) were prepared in order to select the sensitive channel for ethanol response by electronic nose. As shown in Fig. 3a, channels of 10, 11, 12, 13, 14, 15 and 16 were not sensitive to ethanol response, while channels of 1, 2, 3, 4, 5, 6, 7, 8 and 9 exhibited response performances. In contrast, channels of 1, 2 and 5 seemed to be over sensitive, as their signals reached the maximum value at very low concentration, so the ethanol concentration could not be detected within a wide range. By comparison with quadratic function, logarithmic function presented better performance for fitting the channel signals with the ethanol concentration (Table 1). Although channel 3 and channel 4 exhibited almost no difference on the overall performance of ethanol detection. It seems that channel 4 has a slight advantage in the wider range of response signals. However, the correlation *R*^2^ was 0.987 of channel 4 between the ethanol concentrations detected by electronic nose and by HPLC (Fig. 3b), which was lower than the linear model of channel 3 (*R*^2^ = 0.999) (Fig. 3c). Therefore, channel 3 was adopted as an ideal response channel for the on-line detection of ethanol concentration in the following experiments.

**Table 1:** Response analysis of different channels to different ethanol concentrations

Channel	Fitting curve	*R*^2^	Detection limit (g/L)	Range of response signal (V)
1	y = 7.45456 + 0.5329*ln(x−0.09696)	0.959	0.100	4.28–9.95
2	y = 7.28954 + 0.5688*ln(x−0.09619)	0.964	0.109	4.79–9.96
3	y = − 0.657 + 1.40045*ln(x + 2.16909)	0.999	0.261	0.59–6.80
4	y = 0.81894 + 1.41856*ln(x + 0.85516)	0.998	0.211	0.91–8.18
5	y = 5.61217 + 0.96626*ln(x−0.0855)	0.926	0.115	2.21–9.98
6	y = 1.81811 + 1.26012*ln(x + 0.40906)	0.996	0.227	1.25–8.20
7	y = 1.14642 + 1.3473*ln(x + 0.58003)	0.996	0.324	1.01–8.05
8	y = − 2.48811 + 1.77626*ln(x + 5.16912)	0.997	0.324	0.54–6.80
9	y = − 2.23548 + 1.73201*ln(x + 4.61661)	0.997	0.358	0.54–6.79
10	Insensitive	–	–	–
11	Insensitive	–	–	–
12	Insensitive	–	–	–
13	Insensitive	–	–	–
14	Insensitive	–	–	–
15	Insensitive	–	–	–
16	Insensitive	–	–	–

**Fig. 4:**
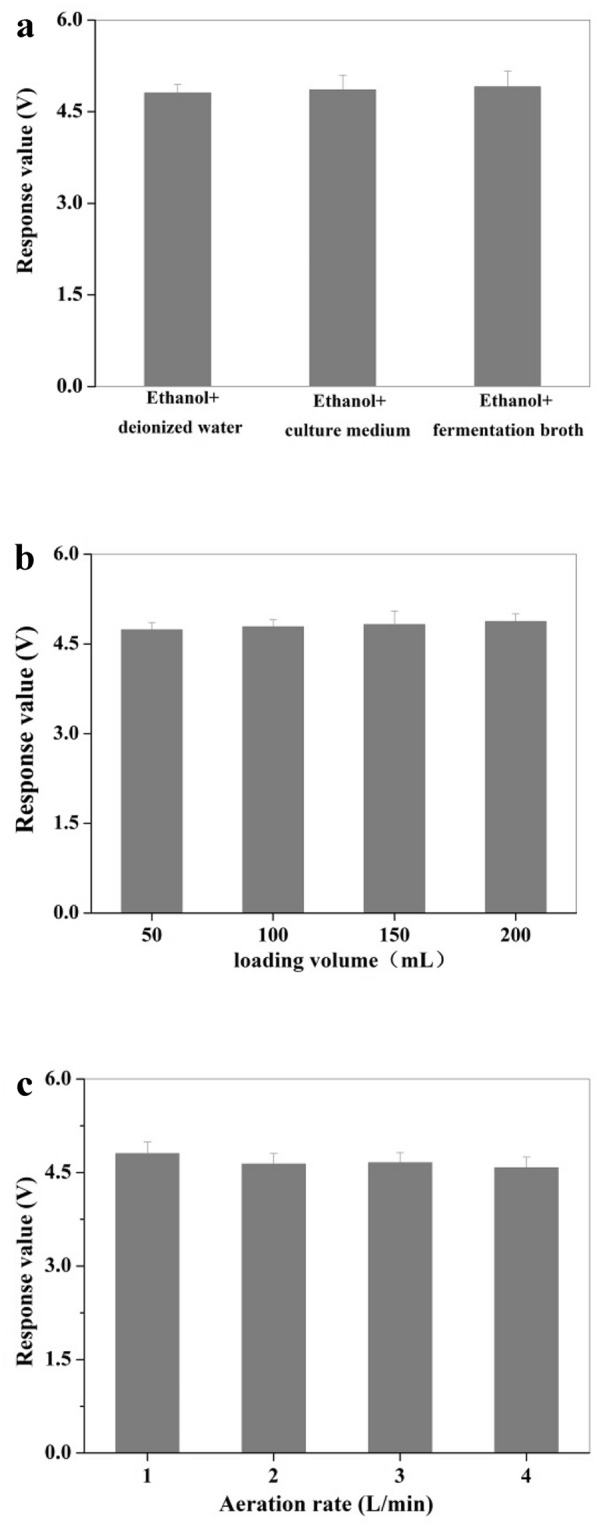
Effects of different ethanol solutions (**a**), loading volume (**b**), and aeration (**c**) on the determination of ethanol concentration in electronic nose-3 channel

The initial fermentation medium contained glucose, KH_2_PO_4_, MgSO_4_, yeast extract, CaCl_2_ and other components; moreover, the composition of fermentation broth was more complex. Besides the product ethanol and biomass, there may also be by-products such as amino acids, organic acids, and glycerol. Therefore, the interferences of components in the fermentation broth on the detection of ethanol concentration by response channel were firstly explored. The results showed that apart from ethanol, other components in the fermentation broth had no significant effect on the response signal of response channel (Fig. 4a). As no aeration was adopted in the fermentation process, an additional detecting device was equipped as shown in Fig. 1, in which the fermentation broth was first pumped into a glass bottle, and then a certain amount of air was aerated at 1 L/min and finally the ethanol content in the off-gas could be detected by the electronic nose. Thus, the effects of the volume of fermentation broth in the glass bottle and the aeration were investigated. The results demonstrated that the amount of liquid filling (50–200 mL) and the aeration (1–4 L/min) had little effect on the response signals (Fig. 4b, c). In the subsequent experiments, 100 mL fermentation broth and 1 L/min aeration were adopted with the consideration of making the vent tube better submerged below the liquid surface, and preventing the liquid splashing into the sample tube of electronic nose.

**Fig. 5:**
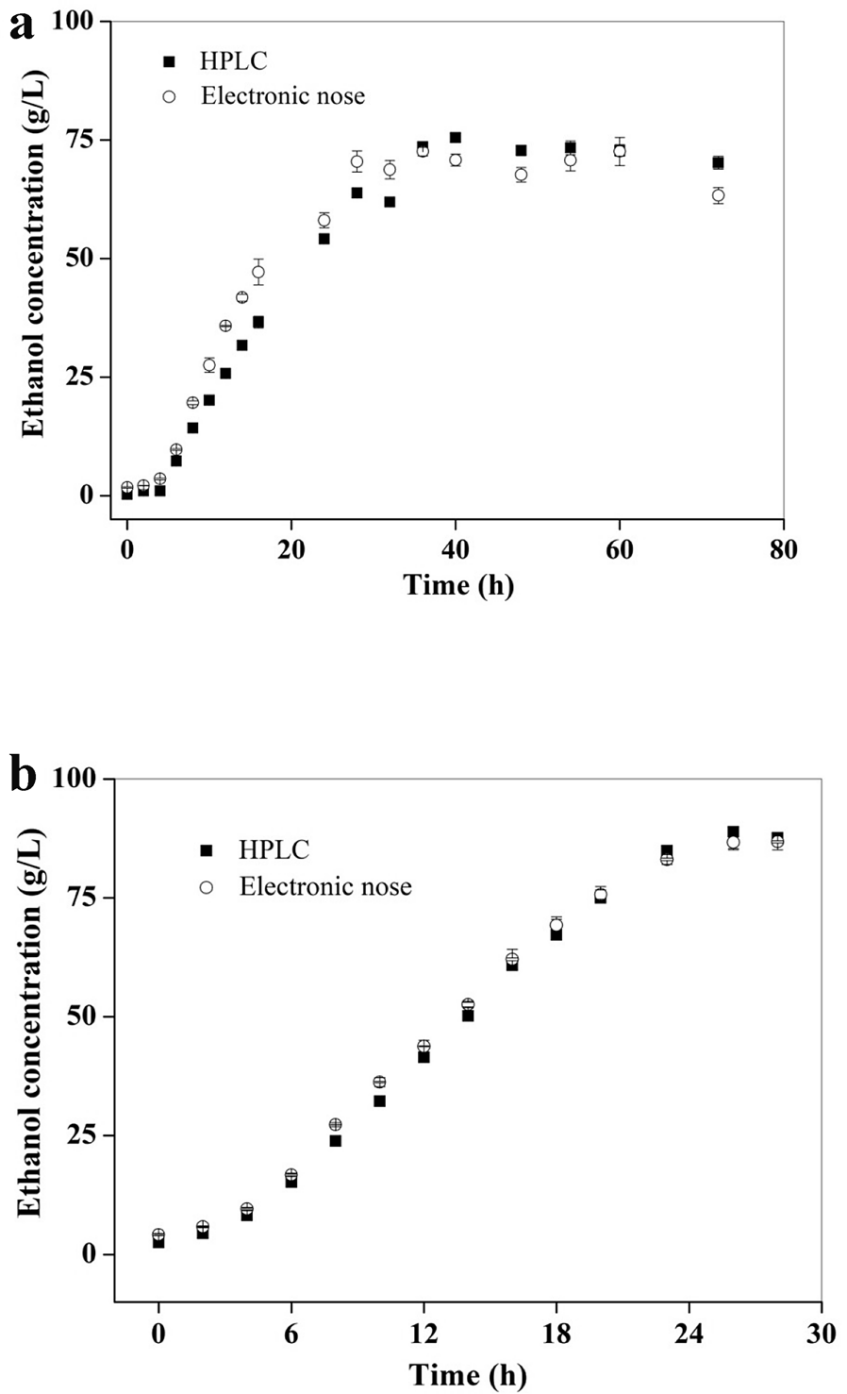
Relationship between the ethanol concentrations determined by electronic nose and by HPLC. **a** Off-line determination of ethanol concentrations in shake-flask fermentation by electronic nose and by HPLC. **b** On-line determination of ethanol concentrations in a 5-L bioreactor fermentation by electronic nose and by HPLC

Apart from comparing ethanol standard solutions of different concentrations (Fig. 3c), the ethanol concentrations in the fermentation broth by electronic nose were further compared with those by HPLC either in a shake-flask or a 5-L bioreactor, demonstrating that the trends of data from electronic nose determination were completely consistent with HPLC (Fig. 5a, b). According to Pearson correlation coefficient analysis, the correlation coefficients of the two groups of data in shake-flask and 5-L bioreactor reached 0.985 and 0.999, respectively, indicating that electronic nose could be used for the on-line detection of ethanol concentration in the fermentation process.

### Development of glucose feeding strategy with the guidance of on-line viable cell sensor and electronic nose

It has been reported that *S. cerevisiae* would suffer from substrate inhibition in the condition of high glucose concentration (Papagianni et al. 2007). In a 5-L bioreactor, the initial glucose concentration of 200 g/L was generally adopted (control batch), which might result in a certain degree of high substrate concentration inhibition. Therefore, a glucose feeding strategy was developed with the guidance of on-line viable cell sensor and electronic nose, so as to verify the feasibility and effectiveness of these sensors in monitoring and regulating the bioprocess as well as achieve the goal of improving ethanol production efficiency.

**Fig. 6:**
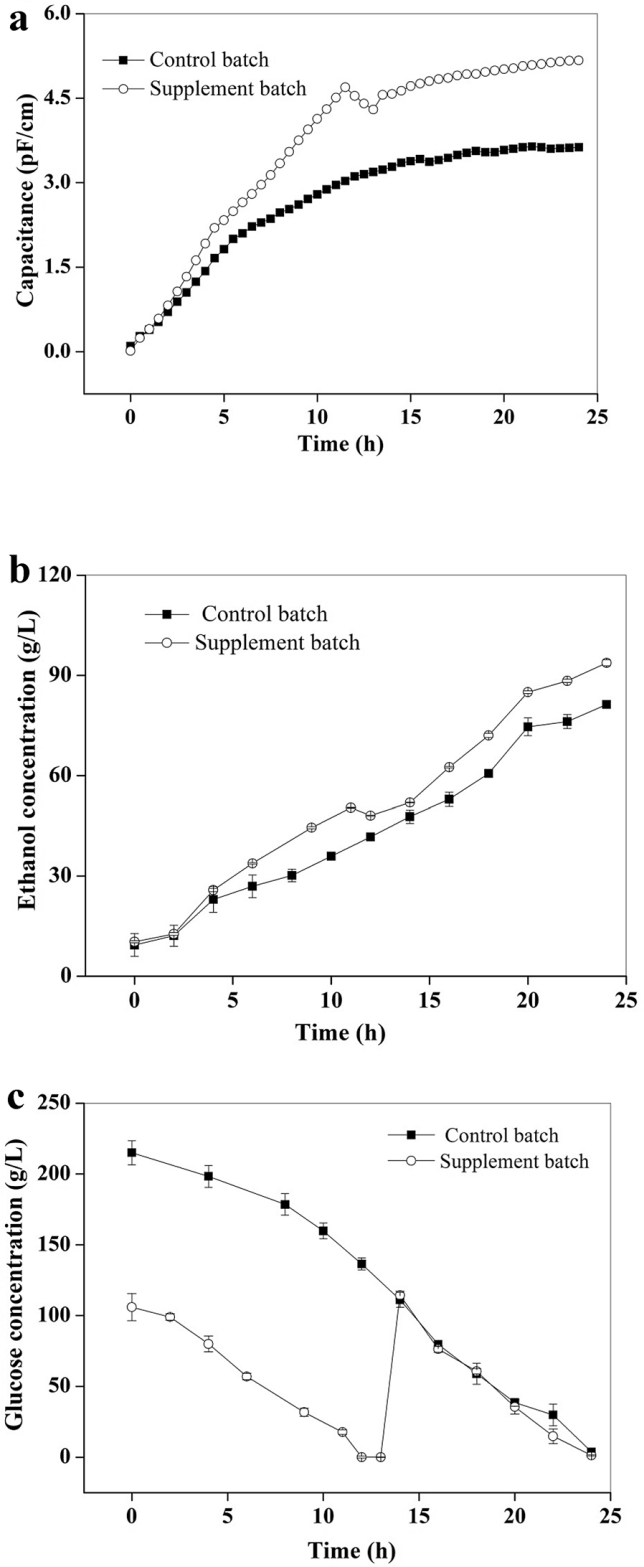
Variation curves of capacitance (**a**), ethanol production (**b**) and glucose consumption (**c**) during 5-L fermentation under the conditions of control batch and supplement batch

When glucose was depleted, the cells would start to consume a small amount of ethanol to maintain basic physiological metabolism (Sabater-Munoz et al. 2020) and simultaneously the number of living cells would decrease. Therefore, in the stepped glucose addition strategy (supplement batch), the initial glucose concentration was 100 g/L, and then the glucose supplement (100 g/L) was guided by two on-line parameters of the capacitance and ethanol content obtained via the viable cell sensor and electronic nose. In the supplement batch, the capacitance value of fermentation broth showed a downward trend between 11.5 and 12.5 h, and the corresponding ethanol concentration also showed a slight decrease (Fig. 6a, b). Therefore, glucose addition was adopted at this time point (12.5 h). Off-line measurement of glucose concentration also verified that glucose was exhausted at about 12 h (Fig. 6c). Comparing with the control batch, the cell growth of the supplement batch was obviously faster, indicating that the inhibition of high substrate concentration could be effectively alleviated by reducing the initial glucose concentration. Notably, although both batches were able to completely deplete the glucose around 24 h (Fig. 6c), the volume of the supplement batch at the end of fermentation was significantly larger than that of the control batch due to the addition of glucose solution. Therefore, when the fermentation volumes of the two batches were standardized to the initial volume, it was obvious that the standardized ethanol concentration of the supplement batch (93.8 g/L) was much higher than that of the control batch (81.3 g/L), with an improvement of 15.4% (Fig. 6b). Meanwhile, the ethanol productivity and yield also increased by 15.9% and 9.0%, respectively (Table 2).

**Table 2:** Comparison of fermentation process between control batch and supplement batch

	Ethanol concentration (g/L initial volume)	Productivity (g/L/h)	Yield (g/g)
Control batch	81.3 ± 0.35	3.19 ± 0.10	0.377 ± 0.015
Supplement batch	93.8 ± 0.66	3.69 ± 0.05	0.411 ± 0.007

The development and application of effective real-time and on-line sensors plays an important role in the optimization of biological processes to improve product concentration, productivity and yield (Alves-Rausch et al. 2014). Biomass which is commonly expressed as OD, DCW, and centrifugal volume, is an important index for cell growth and metabolism. However, these detecting methods are off-line, time-consuming and labor-consuming, more importantly, unable to distinguish living and dead cells. Thus, it is difficult to accurately and real-time reflect the real living cell state in the fermentation process. Flow cytometry is a powerful tool for single cell analysis which can distinguish living cells from dead ones, but it requires complex staining pretreatment (Li et al. 2017) and is generally adopted by off-line approach. Horta et al. (2015) demonstrated that the viable cell sensor is a reliable on-line biomass monitoring tool for Gram-positive and Gram-negative bacteria. Guo et al. (2016) used the on-line capacitance and oxygen uptake rate to control the propanol feed rate to optimize the erythromycin fermentation, which increased the erythromycin titer by 4.0%. In this study, the viable cell sensor was applied into the ethanol fermentation process by *S. cerevisiae* and the changes of biomass in the process were obtained on-line in real-time through the measurement of capacitance value, so as to recognize the growth and metabolic state of cells. As for ethanol detection, the usual determining method is by HPLC (Li et al. 2019; Rehman et al. 2019; Tsai et al. 2020), which is also a kind of off-line detection method with many disadvantages such as high cost, long detection time and use of organic solvent. Although it has been reported that near-infrared spectroscopy or Raman spectroscopy combined with partial least squares regression could simultaneously monitor substrate and product in ethanol fermentation process (Pinto et al. 2016; Nascimento et al. 2017; Legner et al. 2019; Schalk et al. 2017), these methods require expensive near-infrared or Raman instruments and need to build corresponding models for different fermentation systems, which has great limitations in real applications. Electronic nose is widely used in many fields by detecting the content of certain volatile components. Raspagliesi et al. (2020) used electronic nose to analyze volatile organic compounds in the breath of women with suspected ovarian mass and healthy people, and then a diagnostic model was established with a prediction performance of 89% sensitivity and 86% specificity. Herein, electronic nose was applied to the on-line detection of ethanol content in the fermentation process to reflect the change of product concentration in real-time so that we could have a deep understanding of the fermentation characteristics. Finally, a dynamic glucose regulation strategy based on the on-line process sensors was developed, which effectively improved the ethanol concentration, productivity and yield. Hopefully, in future works, more on-line sensors would be adopted integrally in the ethanol fermentation process to realize the full detection of substrates, products, intermediates and cell physiological parameters. And, a more comprehensive understanding of the ethanol fermentation process and more effective strategies for further dynamic regulation could be provided.

## Conclusion

This study introduced the viable cell sensor and electronic nose into the ethanol fermentation process, which could effectively realize the real-time and on-line detection of viable cells and ethanol content in the process. Moreover, with the guidance of these two on-line sensors, a dynamic feeding strategy of glucose was developed. As a result, the ethanol concentration increased by 15.4%, with the improvement of productivity and yield by 15.9% and 9.0%, respectively. The application of advanced sensors in this study can not only deepen the understanding of the cell growth and metabolic characteristics, but also expected to be readily extended to an industrial scale of ethanol fermentation.

## Data Availability

All data generated or analyzed during this study are included in this published article.
